# Protamine1, 2 and Catsper1: sperm quality and fertility indicators in Stallions

**DOI:** 10.1590/1984-3143-AR2025-0040

**Published:** 2025-11-28

**Authors:** Marília Marcolla de Figueiredo, Verônica La Cruz Bueno, Isabele Colla Lazzari Royes, Rodrigo Costa Mattos, Henrique Boll de Araujo Bastos, Sandra Fiala Rechsteiner

**Affiliations:** 1 Laboratório de Reprodução Animal – REPROLAB, Faculdade de Veterinária, Universidade Federal do Rio Grande do Sul, Porto Alegre, RS, Brasil; 2 Programa de Pós-Graduação em Ciências Veterinárias, Universidade Federal do Rio Grande do Sul, Porto Alegre, RS, Brasil; 3 Histologia e Reprodução Equina – HISTOREP, Instituto de Biologia, Universidade Federal de Pelotas, Pelotas, RS, Brasil

**Keywords:** PRM1, PRM2, Catsper1, semen, fertility

## Abstract

The genes identification involved in male reproduction and the evaluation of its functions improve the comprehension about spermatogenesis molecular bases, fertilization, embryos early cleavage, spermatic quality and male infertility. The present study aimed to verify the Protamine1 *(PRM1),* Protamine2 *(PRM2)* and Cation Channel Sperm Associated 1 (*Catsper1)* genes expression into the equine sperm and their relations with the stallions' spermatic quality and fertility. Semen collections were performed in eighteen stallions, which were divided in two groups, based on fertility rates: fertile (with pregnancy rate per cycle ≥ 70%) and subfertile (with pregnancy rate per cycle ≤ 40%). The semen analysis was performed by Computer Assisted Sperm Analysis AndroVision®. The mRNA was extracted from the spermatozoa and the *PRM1*, *PRM2* and *Catsper1* gene expression verification in the spermatic cell was conducted by the qPCR technique. The results present a higher expression of *PRM1* and *Catsper1* in the fertile stallions’ group than subfertile group; there was no correlation of *PRM1* and *PRM2* expression with spermatic quality parameters; there was correlation of the *Catsper1* expression with morphology and motility parameters. Negative correlation was found between the *PRM1/PRM2* ratio, fertility and motility parameters. The present research demonstrates that the *PRM1* and *Catsper1* genes are related to stallions’ fertility and spermatic quality, and they may work as biomarkers.

## Introduction

In equine production, the stallion represents half of the breeding equation. However, a single stallion usually breeds with several mares, so its fertility is a critical factor in the success of a breeding program (Colenbrander et al., 2003). Subfertility or infertility in breeding stallions contributes to low pregnancy rates per cycle and low pregnancy rates per season, resulting in substantial financial losses in the equine industry (Roser, 2011).

Current semen assays are useful predictors of sperm quality and the combination of many sperm function tests improves reliability in fertility estimation, however, they may fail to identify some subfertile stallions (Colenbrander et al., 2003). Studies in humans have revealed a large number of genes that may be involved in male reproductive mechanisms and in the fertilization cascade that could serve as fertility markers (Ostermeier et al., 2005; Aoki et al., 2006; Das et al., 2010). In addition, identifying these genes and the evaluation of their functions would improve our understanding of spermatogenesis molecular basis, fertilization, early embryo cleavage, sperm quality, and male infertility (Ganguly et al., 2013; Bueno et al., 2024).

The spermatic cell may be considered as a specialized and differentiated one, with maximum ergonomics to delivery haploid genome to the oocyte. For that, the spermatic cell minimized its intern volume, it eliminated all of its redundant organelles and silenced all of its non-relevant cellular processes for that task (Boerke et al., 2007). The spermatozoa represented the final stage in the spermatogenic differentiation and they work as a vehicle to transmit the paternal genomic content, transcriptomic and proteomic accumulated to the oocyte (Anton and Krawetz, 2012).

Protamine1 (*PRM1*) and protamine 2 (*PRM2*) genes codify the PRM1 and PRM2 proteins that are inside the nucleus and are specific to the sperm. They bind to DNA to produce a highly condensed chromatin that results in a compact nucleus, with about 5% of the somatic nucleus size (Lee et al., 2011). This strong condensation is necessary to minimize the nuclear volume, improve sperm ergonomics and also to protect genetic material against nucleases, mutagenesis, toxic components and free radicals (Balhorn et al., 2000). The products of *PRM1* and *PRM2* genes are one of the most studied in human sperm, and many studies have related these genes to fertility (Steger et al., 2003; Aoki et al., 2006; Zhang et al., 2006; Steger et al., 2008; Depa-Martynow et al., 2012).

Cation Channel Sperm Associated 1 (*Catsper1*) gene codifies a calcium channel protein that plays a vital role in sperm motility and male fertility (Ren et al., 2001), and is essential for hyperactivated sperm motility (Darszon et al., 2011). Ren et al. (2001) knocked out *Catsper1* gene from mice, which resulted in poor sperm motility and infertility. Catsper channels are also necessary to allow the sperm to penetrate the zona pellucida (Ren et al., 2001). The general trigger for Catsper opening is alkaline depolarization caused by the change in the oviduct ionic environment (Carlson et al., 2003). The sperm contact with the alkaline environment increases intracellular pH that activates Catsper channels (Leemans et al., 2019). Nikpoor et al. (2004) found decreased Catsper gene expression in subfertile men with low sperm motility and conclude that this may contribute to the diagnosis and treatment of infertility cases attributed to problems in sperm motility. The presence of *Catsper1* mRNA was identified in stallion sperm (Loux et al., 2010), but it is still unclear when these channels open in stallion sperm. The failure in stimulation or in hyperactivate motility of equine sperm under standard capacitation conditions for other species may be related to species-specific differences in the presence or function of Catsper channels (Loux, 2013). Research on human fertility and that of other mammals has made significant progress in recent years. However, there is still limited research on the molecular mechanisms influencing stallion fertility. The present study aimed to investigate the expression of *PRM1*, *PRM2* and *Catsper1* genes in equine sperm and their relationship with sperm quality and fertility in stallions.

## Methods

The experiments were carried out at the Animal Reproduction Laboratory (REPROLAB) of the Veterinary College in the Federal University of Rio Grande do Sul.

The present experiment was conducted during the southern hemisphere breeding season (November to March) in the south of Brazil (30° S, 51° W). The study was approved by the Committee of Ethical Use in Animal Experimentation at Federal University of Rio Grande do Sul (UFRGS), Brazil (protocol number 38666).

### Animals

Eighteen criollo stallions aged between 3 to 24 years old (13,7 ± 6,8 years old) and weighing between 450 and 500 kg were used, all animals were healthy and with an average body condition score 3.5 (scale 1 to 5) (Malschitzky et al., 2001), they were fed daily with concentrate and alfalfa hay, with water and mineral salt *ad libitum*. One ejaculate was collected from each stallion that came from breeding centers, during the same breeding season. All stallions were in a routine of semen collecting three times a week at the breeding centers. The fertility of each stallion was defined by calculating the pregnancy rate per cycle using data of 30 mares/stallion, in the same reproductive season in which samples were collected. Only data from natural breeding and artificial insemination with fresh semen were analyzed according to Bueno et al., (2024).

The rates of the reproductive season in which the collections were performed were considered. Fertility rates ranged from 20 to 90%. Based on fertility rates the stallions were divided into two groups: fertile (with pregnancy rate per cycle ≥ 70%, n = 11) and subfertile (with pregnancy rate per cycle ≤ 40%, n = 7).

### Semen analysis

The semen collected was realized in an artificial vagina, Hannover model. After collecting, the samples were stored in Falcon tubes and sent to the Animal Reproduction Laboratory (REPROLAB) of the Veterinary College in the Federal University of Rio Grande do Sul, with a maximum transport limit of 2 hours in a transport box Botuflex® (Botupharma, Botucatu, SP, Brazil) without refrigeration or dilution. Each sample was separated in two fractions, one to evaluate the semen parameters and the other to later mRNA extraction.

The sperm concentration was evaluated with a hemocytometer (Neubauer chamber) (Brito, 2007). The evaluations of Total Motility (%) (TM); Progressive Motility (%) (PM); Fast Motility (%) (FM); Slow Motility (%) (SM); Local Motility (%) (LM); Average Path Velocity (VAP, μm/s); Straight Line Velocity (VSL, μm/s); Curvilinear Velocity (VCL, μm/s); Amplitude of Lateral Head Displacement (ALH, μm); Beat Cross Frequency (BCF, Hz); Straightness (STR, %) (VSL/VAP); Linearity (LIN, %), (VSL/VCL) was performed using the Computer Assisted Sperm Analysis (CASA) system, Tiefenbach, Germany, AndroVision®, Minitube). The sperm sample was placed in a disposable 4-chamber slide (20 microns in depth; Leja Products B.V., Amsterdam, The Netherlands). The system AndroVision® classified sperm based on motility patterns and velocity parameters, following a structured decision tree. The motility parameters assessed included Total Motility (TM), representing sperm exhibiting any movement; Progressive Motility (PM), defined by a curvilinear velocity (VCL) greater than 40.00 μm/s and a straight-line velocity (VSL) above 10.00 μm/s; Circular Motility (CM), characterized by sperm exhibiting circular movement with a radius between 10.00 and 60.00 μm and a rotation value above 0.70; Local Motility (LM), referring to motile sperm that do not show progressive movement, with VCL below 40.00 μm/s and VSL under 10.00 μm/s; Fast Motility (FM) and Slow Motility (SM), where slow sperm presented VCL values below 120.00 μm/s, whereas fast sperm displayed VCL values equal to or greater than 120.00 μm/s; and Immotile Sperm (IS), defined by an amplitude of lateral head displacement (ALH) below 4.00 μm and a beat cross frequency (BCF) lower than 4.00 Hz. Additionally, other kinematic parameters such as Average Path Velocity (VAP), Straightness (STR = VSL/VAP), and Linearity (LIN = VSL/VCL) were recorded. The CASA system operated at a frame rate of 30 images per second at 60 Hz, capturing particles within a size range of 4 to 75 μm^2^. This CASA setup enabled a detailed and quantitative assessment of sperm motility, ensuring precise differentiation between motility patterns and providing accurate kinematic data for reproductive analysis.

For the plasma membrane physical integrity analysis, 400 μl semen was incubated with 3μl of propidium iodide (PI) and 2μl of carboxyfluorescein diacetate (CFDA) at 37°C for eight minutes. The sample was evaluated by epifluorescence microscopy at 1000x magnification. A total amount of 100 cells per sample were evaluated. The cells stained green were considered intact and cells stained partially or totally stained in red are considered damaged sperm (Garner et al., 1986).

The plasma membrane functional integrity was evaluated by hyposmotic test, in which 200 μL of distilled water were added to 100 μL of semen and incubated at 37º C for eight minutes. After that, the samples were analyzed in a phase contrast microscope at 400x magnification. A hundred cells were analyzed and considered positive reacted (HOST +) when the tail was coiled, the numbers analyzed were discounted the number of coiled tails of the morphology (Lagares et al., 2000).

Panoptic® kit (Laborclin) was used to evaluate sperm morphology, the semen smear slide was dipped for 20 seconds in the dyes, and immediately after drying the slide was taken to a microscope in an immersion objective (1000x) for analysis, 100 sperm cells were counted from each sample (CBRA, 2013; Segabinazzi et al., 2017).

The semen fraction which would be used later for extracting mRNA was centrifuged at 600x g for 10 minutes. This procedure was repeated 3 times. After each centrifugation, the supernatant was discarded and the pellet was resuspended in PBS medium. After the third centrifugation the pellet was resuspended in 2 ml of RNA later^®^ (Life Technologies) in free RNA cryotube and stored in a freezer at -80ºC to later mRNA extraction.

### Extraction of mRNA

The mRNA extraction was made by the Commercial Kit SVRNA Total Isolation System® (Promega, Madison, WI, USA), according to the manufacture instructions. The samples RNA concentration was evaluated with Nanovue and they were stored in a freezer at -80ºC.

After the extraction, the mRNA was quantified by spectrophotometry (NanoVue Plus, GE Healthcare). Only RNA samples with 260/280 ratio between 1.9 and 2.1 and 260/230 ratio >2.0 were used for the analysis.

### cDNA and qPCR

Reverse transcription of mRNA to cDNA was performed using the GoScript Reverse Transcription System (Promega, Madison, WI, USA), according to the manufacturer instructions. The cDNA concentration of the samples was evaluated with NanoVue Plus, GE Healthcare and the samples were stored in a freezer at -20°C.

The expression of *PRM1*, *PRM2* and *Catsper1* genes in the sperm cell was verified by qPCR technique. The amplification of the cDNA was performed by a primer designed specifically for an amplicon (sequence of interest) using the BRYT Green fluorophore from the kit qPCR MasterMix 2XGoTaq® (Promega, Wisconsin, WI, USA). The primers were obtained from Integrated DNA Technologies (IDT^®^) and the sequences used are listed in Table 1.

**Table 1:** Details of the sequences used for quantitative real-time polymerase chain reaction amplification of mRNA from stallion’s sperm cells.

**Gene**	**Primer**	**Annealing temperature**	**References**
*PRM1*	F:5’-CGATAGTGCACGAGACAGCAA - 3’R:5’-ATGGTGGCATTTTCAAGATGTG - 3’	58°C	(Ing et al., 2014)
*PRM2*	F:5’-CACACCCCGGAGAATATCGA-3’R: 5’-CGTCGTCTGTAGCGGTAGTAGCT-3’	58°C	(Ing et al., 2014)
*Catsper1*	F:5’-GGTGCTGCGAGCCTTGTT-3’R: 5’- TGAATATGGTCGTGAAGATGTTCTG-3’	60°C	(Ing et al., 2014)
*β-actin*	F:5’-CGACATCCGTAAGGACCTGT - 3’R:5’-GTGGACAATGAGGCCAGA AT - 3’	60°C	(Coyne et al., 2009)

*PRM1*: Protamine1; *PRM2*: Protamine 2; *Catsper1*: Cation Channel Sperm Associated 1; F: forward primer; R: reverse primer.

Relative quantitation was performed and the mRNA levels of the target genes (*PRM1*, *PRM2* and *Catsper1*) were normalized against *β-actin* mRNA levels. The endogenous *β-actin* gene was used to normalize the amount of RNA added in the reactions. To determine the assay amplification efficiency a 5x serial dilution was performed, starting at a concentration of 242,5 ng/ml to 0,0024 ng/ml, with one sample for each gene used to perform the standard curve. For results normalization and accuracy in comparing gene expression between samples, the RNA concentration of 1ng/ml was used for all samples. Threshold cycle (CT) method of comparison was used to calculate the relative mRNA expression (2 ^−ΔΔCT^).

The program profile used for amplification was 95 °C for 2 minutes followed by 40 cycles of denaturation at 95°C for 3 seconds, annealing for 30 seconds and extension at 60 °C for 30 seconds. The amplification was performed using the thermal cycler StepOne™ Real-Time PCR System (Applied Biosystems, Foster City, CA, USA), and the data were processed using Step One Plus^TM^ Software v2, 3 (Applied Biosystems, Foster City, CA, USA).

### Statistical analysis

Data were analyzed using GraphPad Prism 10.3.1 software, and the normality of the results were evaluated using the Shapiro-Wilk test and Anderson-Darling test. Animals were divided into two groups: group Fertile and group Subfertile. Groups were considered as an independent variable, and gene expression of *PRM1*, *PRM2*, *Catsper1* and seminal parameters (TM, PM, CM, FM, SM, LM, IS, VAP, VSL, VCL, ALH, BCF, STR, LIN), plasma membrane integrity and functionality, major and minor defects and *PRM1/PRM2* ratio, were considered as dependent variables. Dependent variables that follow a normal distribution were performed using one-way ANOVA, followed by Tukey’s t-test. Variables that did not follow a normal distribution were evaluated using the non-parametric Mann Whitney test. Differences with P < 0.05 were considered significant. Pearson’s correlation coefficient was applied to verify the relationship between all dependent variables in the study. With a significance level of P < 0.05, correlations with values from 0,40 to 0,60 were considered as correlations of moderate intensity and those with values > 0,60 were considered as correlations of strong intensity.

## Results

The results of semen analysis, mean and standard deviation of the Fertile and Subfertile groups, are shown in Table 2. Some analyzed variables, TM, PM, Immotile, VSL, VAP, and BCF showed differences between the two groups.

**Table 2:** Sperm quality and kinetic parameters analyzed in groups of Fertile and Subfertile stallions (Mean ± SD).

**Variable**	**Fertile (mean ± SD)**		**Subfertile (mean ± SD)**	
Sperm concentration (x 10^6^/mL)	106.50	± 0.70	180.0	± 212.13
HOST (%)	69.00	± 8.48	52.50	± 2.12
Integrity of plasma membrane by fluorescence(%)	90.00	± 9.89	49.50	± 19.09
Normal morfology(%)	73.50	± 19.09	69.65	± 0.91
Minor defects (%)	8.25	± 1.06	8.80	± 1.13
Major defects(%)	18.25	± 18.03	21.65	± 1.90
Fertility(%)	70.50	± 0.70^^a^^	30.00	± 14.14^^b^^
TM (%)	76.50	± 4.95^^a^^	33.65	± 9.26^^b^^
PM (%)	49.50	± 16.97^a^	16.45	± 2.61^b^
FM(%)	9.75	± 6.29	3.30	± 4.66
SM (%)	36.45	± 23.26	10.80	± 2.54
LM(%)	27.40	± 21.35	17.25	± 6.57
Immotile(%)	14.10	± 17.11^^a^^	66.35	± 9.26^^b^^
VCL(µm/s)	122.23	± 22.06	66.29	± 25.03
VSL(µm/s)	43.78	± 8.48^^a^^	25.47	± 7.16^^b^^
VAP(µm/s)	54.25	± 6.01^^a^^	32.21	± 8.89^^b^^
LIN(%)	0.32	± 0.00	0.32	± 0.22
BCF(Hz)	18.34	± 1.73^^a^^	9.83	± 1.35^^b^^
ALH(µm)	1.22	± 0.17	0.66	± 0.20

**Figure 1:**
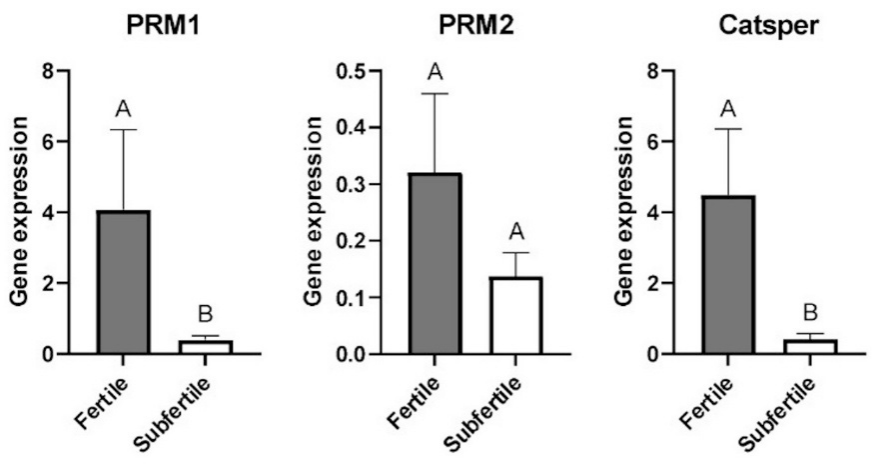
Mean (± SEM) of *PRM1*, *PRM2* and *Catsper1* gene expression of sperm from fertile and subfertile stallions. Different letters (A, B) indicate significant difference (*P* < 0.05).

TM: Total Motility; PM: Progressive Motility; FM: Fast Motility; SM: Slow Motility; LM: Local Motility; VCL: Curvilinear Velocity; VSL: Straight Line Velocity; VAP: Average Path Velocity; LIN: Linearity (VSL/VCL); BCF: Beat Cross Frequency; ALH: Amplitude of Lateral Head Displacement. Different letters (a.b) indicate significant differences (*P*<0.05). The differences between the groups to the expression of *PRM1* (*P* = 0.039) and *Catsper1* (*P*=0.046) genes are shown in Figure 1. We found a trend between the groups to express *PRM2* gene (*P* = 0.08), as shown in Figure 1.

Correlation results between *PRM1/PRM2* ratio and sperm quality data with significative difference (*P* < 0.05) are shown in Table 3. The results of the correlations found between *Catsper1* gene expression and sperm kinetic analysis with significative difference (*P* < 0.05) are shown in Table 4.

**Table 3:** Pearson coefficient of correlation between seminal quality and kinetic parameters and the *PRM1/PRM2* ratio in stallions, with significative difference (*P* < 0.05).

**Membrane integrity**		**Fertility**	**Total motility**	**Immotile**	**VCL**	**VAP**	**BCF**	**ALH**
R	-0.85	-0.81	-0.89	0.90	-0.85	-0.72	-0.92	-0.85
P	0.00	0.01	0.00	0.00	0.00	0.04	0.00	0.00

VCL: curvilinear velocity (μm/s); VAP: average path velocity (μm/s); BCF: beat cross frequency (Hz); ALH: amplitude of lateral head displacement (μm).

**Table 4:** Pearson coefficient of correlation between seminal quality and kinetic parameters and *Catsper1* gene in stallions, with significative difference (*P* < 0.05).

	**Minor defects**	**PM**	**FM**	**BCF**
R	-0.69	0.86	0.96	0.68
P	0.03	0.00	0.00	0.03

PM: progressive motility (%); LM: local motility (%); FM: fast motility (%); LIN: linearity (%); BCF: beat cross frequency (Hz).

## Discussion

The present study demonstrated results on the gene expression of *PRM1*, *PRM2* and *Catsper1* in sperm of fertile and subfertile stallions. Several studies in men related protamines mRNA levels to fertility and demonstrated the importance of this genes (Steger et al., 2003; Aoki et al., 2006; Zhang et al., 2006; Steger et al., 2008).

In the present study, *PRM1* mRNA levels were higher in the group of fertile stallions compared to subfertile stallions. These results agree with the findings of Pardede et al. (2022) that demonstrated a higher expression of *PRM1* mRNA and more abundant PRM1 protein in high fertile bulls than in low fertile bulls; and with the findings of Depa-Martynow et al. (2012) that found a correlation between mRNA levels and PRM1 protein concentration, where both would be increased in sperm from men who were successful in vitro fertilization.

The results of this research demonstrate the difference in expression of *PRM1* between the groups; the changes in protamine expression probably originate from spermatogenesis as demonstrated by Steger et al. (2003) who observed a significant decrease of mRNA levels in *PRM1* in the groups of patients with compromised spermatogenesis. According to Paradowska-Dogan et al. (2014), the protamine’s expression in stallions as well as in humans constitutes a checkpoint of spermatogenesis and the protamine mRNA level may reflect spermatogenesis quality and sperm fertilization capacity.

The *PRM1* expression findings of the present study disagree with Ing et al. (2014) who submitted stallion ejaculates to density gradient centrifugation and created two groups, one with the dense sperm and the other with the less dense sperm. They found no differences in *PRM1* mRNA levels between the two groups. We believe that this divergence between studies is due to the methodology used, since the sperm selection of the same ejaculate does not reflect problems in the spermatogenesis. Another work that disagrees with the present findings is the one of Aoki et al. (2006) who evaluated mRNA levels and PRM1 protein concentrations in infertile men and found higher *PRM1* mRNA levels related to lower PRM1 protein concentrations in infertile men, the hypothesis mentioned is that there was an error during translation with consequent retention of mRNA.

There is no consensus between protamine levels and sperm kinetics correlation (Kempisty et al., 2007; Depa-Martynow et al., 2012; Jodar et al., 2013; Ganguly et al., 2013; Hamad, 2019). The present study found no positive correlation between *PRM1* gene expression and sperm quality evaluations. These findings are consistent with researches that found no correlation between *PRM1* expression and sperm quality (Hamad, 2019). Jodar et al. (2013) found no significant differences between protamine gene levels in sperm of asthenozoospermic in fertile men compared to normozoospermic infertile men. According to these authors, the results suggest that differences in protamines quantity are more correlated with fertility than with motility parameters. Other studies have shown a relationship between expression and sperm motility. Ganguly et al. (2013) compared *PRM1* mRNA levels in bulls with high and low sperm motility. They observed high levels of *PRM1* expression in good quality semen compared with the low quality. Kempisty et al. (2007) found a higher level of *PRM1* mRNA in sperm from normozoospermic men compared to asthenozoospermic men. Depa-Martynow et al. (2012) found a positive correlation with sperm quality and *PRM1* mRNA levels.

The present research did not identified differences in *PRM2* expression between groups. These findings are according to the study that evaluated the *PRM2* mRNA levels in fertile and infertile men and found higher levels, but with no statistical difference in fertile men (Aoki et al., 2006), and agree to Avendaño et al. (2008) that found no differences in *PRM2* mRNA between the fertile men groups in comparison to the infertile ones and also agree from studies that could not find any difference in *PRM2* mRNA level among the analyzed groups (Steger et al., 2003; Ganguly et al., 2013).

In this research wasn’t verified correlations between *PRM2* and sperm quality, which agrees with Ganguly et al. (2013) that compared the*PRM2* mRNA levels in bulls with high and low sperm motility and didn’t find relation to sperm quality. These current results differ from the researches that had found positive correlation between the sperm quality and *PRM2* mRNA levels (Depa-Martynow et al., 2012; Hamad, 2019) and from Kempisty et al. (2007) that had found higher levels of *PRM2* mRNA in normozoospermic men’s sperm in relation to the asthenozoospermic ones.

Most protamine researches were conducted in humans, and in these studies, subfertility was correlated with abnormal histone persistence (Zhang et al., 2006; Hamad, 2019) or an abnormal *PRM1/PRM2* ratio in protein concentrations in sperm and in mRNA levels in spermatids and sperm (Steger et al., 2003; Aoki et al., 2006; Depa-Martynow et al., 2012; Rogenhofer et al., 2013). In humans, protein expression has a PRM1/PRM2 ratio of approximately 1,0 (Aoki et al., 2005). Sperm from infertile men show altered *PRM1/PRM2* ratio or undetectable *PRM2* in mature sperm (Sharma and Agarwal, 2011), with a *PRM1/PRM2* >1 ratio (Carrell and Liu, 2001) and embryos derived from sperm deficient in *PRM2* had their development affected (Cho et al., 2003). Several authors (Carrell and Liu, 2001; Aoki et al., 2005; Rogenhofer et al., 2013) have suggested the protamine ratio could be used as a potential clinical parameter for evaluating fertility in humans. Paradowska-Dogan et al. (2014) correlated the *PRM2/PRM1* ratio in stallions’ testis with different fertility rates and concluded that protamine gene levels in equine may reflect spermatogenesis quality and sperm fertilizing capacity. The current study found a negative correlation between the *PRM1/PRM2* ratio with fertility, which agrees with other studies that identified a higher value of the *PRM1/PRM2* ratio in subfertile groups.

The present study found correlation between *PRM1/PRM2* ratio and sperm quality data, which agrees with the findings in men of Rogenhofer et al. (2013) and Depa-Martynow et al. (2012) and disagrees with studies that found no correlations between the *PRM1/PRM2* ratio with sperm number, motility and sperm morphology (Hamad et al., 2017; Hamad, 2019).

In the present work it was found differences in the levels of *Catsper1* gene expression between groups, where the expression was higher in the fertile horses group compared to the subfertile one. The *Catsper1* gene encodes a calcium channel protein that is unique to other sperm (Loux et al., 2013). This protein is necessary for normal sperm motility and sperm penetration into the zona pellucida (Ren et al., 2001). Mice without any *Catsper* isoform are infertile (Ren et al., 2001; Qi et al., 2007), and mutations in human *Castper1* genes are associated with infertility (Avenarius et al., 2009). The human *Catsper* gene is a potential target for male infertility screening (Ren et al., 2001). This gene was identified in stallion sperm (Loux et al., 2013) and was studied in stallion semen submitted to density gradient selection where it was found lower *Catsper1* mRNA concentrations in dense sperm (Ing et al., 2014).

The higher *Catsper1* expression observed in the present study aligns with findings by Al-Msaid and Al-Sallami (2018), who reported reduced Catsper1 protein expression in normospermic, asthenozoospermic, and oligozoospermic infertile men compared to fertile normospermic men. In this study, several correlations were identified between *Catsper1* expression and sperm quality parameters, including positive correlations with progressive, circular, and slow motility, as well as morphology. This is consistent with Al-Msaid and Al-Sallami's (2018) observation of a positive correlation between *Catsper1* expression and sperm concentration, progressive motility, and normal sperm morphology in infertile men without a diagnosed cause. A group of researchers supplemented young and old mice with selenium (Mohammadi et al., 2009) and vitamin E (Mohammadi et al., 2013) to evaluate their effects on *Catsper1* and *2* gene expression and sperm parameters. They observed an increase in *Catsper* gene expression, particularly *Catsper1*, and improvements in sperm parameters. The effects were more pronounced in older mice. The researchers concluded that both selenium and vitamin E treatments boosted *Catsper1* and *2* gene expression and improved sperm quality (including concentration, morphology, motility, and viability rates). Later, the same group treated adult mice with lead and mercury (Mohammadi et al., 2018) to assess their effects on *Catsper1* and *2* gene expression in seminiferous tubules and sperm parameters. They found degeneration in seminiferous tubules, reduced sperm quality, and decreased expression of both *Catsper1* and *2* genes. This pattern of improvement with supplementation and decline with toxic exposure aligns with the present study's findings, which showed correlations between *Catsper1* expression and sperm morphology, progressive motility, slow motility, and beat-cross frequency (BCF).

Tamburrino et al. (2014) observed lower levels of *Catsper1* protein expression in asthenozoospermic men compared to normozoospermic men and found that this expression is strongly correlated with the percentage of progressive motility in semen samples. They demonstrated that an in vitro treatment with Catsper inhibitors strongly affected sperm kinetic parameters and reduced total, progressive and rapid motility. The authors suggested there is a strong indication of the connection between Catsper channel expression and function and sperm motility, which agrees with the findings of the progressive motility correlation of the present experiment.

## Conclusion

In conclusion, *PRM1*, *PRM2* and *Catsper1* genes are expressed in equine sperm and our results suggest a potential association with fertility and sperm quality in stallions. Protamine 1 is probably more related to spermatogenesis problems, while *Catsper1* gene expression seems to be more related to sperm quality, *PRM1/PRM2* ratio has been shown to be positively related to sperm quality data. We suggest that *PRM1* and *Catsper1* genes may be used as biomarkers of fertility and sperm quality on stallion semen.

## References

[B001] Al-Msaid HLF, Al-Sallami ASM (2018). Study of catsper1 protein levels in unexplained andidiopathic infertile men. Int J Pharm Qual Assur.

[B002] Anton E, Krawetz SA (2012). Spermatozoa as biomarkers for the assessment of human male infertility and genotoxicity. Syst Biol Reprod Med.

[B003] Aoki VW, Liu L, Carrell DT (2005). Identification and evaluation of a novel sperm protamine abnormality in a population of infertile males. Hum Reprod.

[B004] Aoki WA, Liu L, Carrell DT (2006). A novel mechanism of protamine expression deregulation highlighted by abnormal protamine transcript retention in infertile human males with sperm protamine deficiency. Mol Hum Reprod.

[B005] Avenarius MR, Hildebrand MS, Zhang Y, Meyer NC, Smith LLH, Kahrizi K, Najmabadi H, Smith RJH (2009). Human male infertility caused by mutations in the CATSPER1 channel protein. Am J Hum Genet.

[B006] Avendaño C, Franchi A, Oehninger SC (2008). Human sperm mRNA: differential expression between fertile and infertile men and maintenance of transcripts after fertilization. Fertil Steril.

[B007] Balhorn R, Brewer L, Corzett M (2000). DNA condensation by protamine and arginine-rich peptides: analysis of toroid stability using single DNA molecules. Mol Reprod Dev.

[B008] Boerke A, Dieleman SJ, Gadella BM (2007). A possible role for sperm RNA in early embryo development. Theriogenology.

[B009] Brito LFC (2007). Evaluation of stallion sperm morphology. Clin Tech Equine Pract.

[B010] Bueno VLC, Bastos HBA, Centeno LA, Kretzmann NA, Mattos RC, Rechsteiner SF (2024). PLCζ. WBP2NL and TNF-α expression in spermatozoa is associated with stallion fertility and seminal quality?. Anim Reprod.

[B011] Carlson AE, Westenbroek RE, Quill T, Ren D, Clapham DE, Hille B, Garbers DL, Babcock DF (2003). CatSper1 required for evoked Ca^2+^ entry and control of flagellar function in sperm. Proc Natl Acad Sci USA.

[B012] Carrell DT, Liu L (2001). Altered protamine 2 expression is uncommon in donors of known fertility, but common among men with poor fertilizing capacity, and may reflect other abnormalities of spermiogenesis. J Androl.

[B013] CBRA (2013). Manual para exame andrológico e avaliação de sêmen animal..

[B014] Cho C, Jung-Ha H, Willis WD, Goulding EH, Stein P, Xu Z, Schultz RM, Hecht NB, Eddy EM (2003). Protamine 2 deficiency leads to sperm DNA damage and embryo death in mice. Biol Reprod.

[B015] Colenbrander B, Gadella BM, Stout TE (2003). The predictive value of semen analysis in the evaluation of stallion fertility. Reprod Domest Anim.

[B016] Coyne MJ, Cousin H, Loftus JP, Johnson PJ, Belknap JK, Gradil CM, Black SJ, Alfandari D (2009). Cloning and expression of ADAM-related metalloproteases in equine laminitis. Vet Immunol Immunopathol.

[B017] Darszon A, Nishigaki T, Beltran C, Treviño CL (2011). Calcium channels in the development, maturation, and function of spermatozoa. Physiol Rev.

[B018] Das PJ, Paria N, Gustafson-Seabury A, Vishnoi M, Chaki SP, Love CC, Varner DD, Chowdhary BP, Raudsepp T (2010). Total RNA isolation from stallion sperm and testis biopsies. Theriogenology.

[B019] Depa-Martynow M, Kempisty B, Jagodziński PP, Pawelczyk L, Jedrzejczak P (2012). Impact of protamine transcripts and their proteins on the quality and fertilization ability of sperm and the development of preimplantation embryos. Reprod Biol.

[B020] Ganguly I, Gaur GK, Kumar S, Mandal DK, Kumar M, Singh U, Kumar S, Sharma A (2013). Differential expression of protamine 1 and 2 genes in mature spermatozoa of normal and motility impaired semen producing crossbred Frieswal (HF × Sahiwal) bulls. Res Vet Sci.

[B021] Garner DL, Pinkel D, Johnson LA, Pace MM (1986). Assessment of spermatozoal function using dual fluorescent staining and flow cytometric analyses. Biol Reprod.

[B022] Hamad M, Shelko N, Montenarh M, Hammadeh ME (2017). The impact of cigarette smoking on protamines 1 and 2 transcripts in human spermatozoa. Hum Fertil.

[B023] Hamad MF (2019). Quantification of histones and protamines mRNA transcripts in sperms of infertile couples and their impact on sperm’s quality and chromatin integrity. Reprod Biol.

[B024] Ing NH, Forrest DW, Love CC, Varner DD (2014). Dense spermatozoa in stallion ejaculates contain lower concentrations of mRNAs encoding the sperm specific calcium channel 1, ornithine decarboxylase antizyme 3, aromatase, and estrogen receptor alpha than less dense spermatozoa. Theriogenology.

[B025] Jodar M, Selvaraju S, Sendler E, Diamond MP, Krawetz AS (2013). The presence, role and clinical use of spermatozoal RNAs. Hum Reprod Update.

[B026] Kempisty B, Depa-Martynow M, Lianeri M, Jedrzejczak P, Darul-Wasowicz A, Jagodzinski P (2007). Evaluation of protamines 1 and 2 transcript contents in spermatozoa from asthenozoospermic men. Folia Histochem Cytobiol.

[B027] Lagares MA, Petzoldt R, Sieme H, Klug E (2000). Assessing equine sperm-membrane integrity. Andrologia.

[B028] Leemans B, Stout TA, De Schauwer C, Heras S, Nelis H, Hoogewijs M, Van Soom A, Gadella BM (2019). Update on mammalian sperm capacitation: how much does the horse differ from other species?. Reproduction.

[B029] Lee TL, Cheung AHH, Rennert OM, Chan WY, Zini A, Agarwal A (2011). Sperm chromatin..

[B030] Loux SC, Crawford KR, Ing NH, González-Fernández L, Macías-García B, Love CC, Varner DD, Velez IC, Choi YH, Hinrichs K (2013). CatSper and the relationship of hyperactivated motility to intracellular calcium and pH kinetics in equine sperm. Biol Reprod.

[B031] Loux SC, Crawford KR, Norris JD, Ing NH, Reynolds EA, Love CC, Varner DD, Velez IC, Dangott LJ, Thornton ER, Choi YH, Hinrichs K (2010). The CatSper ion channel and hyperactivated motility of horse spermatozoa. Anim Reprod Sci.

[B032] Loux SC (2013). Hyperactivated motility of stallion spermatozoa.

[B033] Malschitzky E, Schilela A, Meirelles LS, Mattos ALG, Gregory RM, Mattos RC (2001). Artificial photoperiod in pregnant mares and its effect on pregnancy length and postpartum reproductive performance. Pferdeheilkunde.

[B034] Mohammadi S, Movahedin M, Mowla SJ (2009). Up-regulation of CatSper genes family by selenium. RB&E..

[B035] Mohammadi S, Jalali M, Nikravesh MR, Fazel A, Ebrahimzadeh A, Gholamin M, Sankian M (2013). Effects of Vitamin E treatment on CatSper genes expression and sperm quality in the testis of the aging mouse. Iran J Reprod Med.

[B036] Mohammadi S, Gholamin M, Mohammadi M, Mansouri A, Mahmoodian R, Attari S, Kebriaei SM, Zibaei B, Roshanaei M, Daneshvar F, Khandehro M, Khodadadegan MA, Delshad A, Mohammadzadeh F, Peyvandi M, Ghayour-Mobarhan M, Tavallaie S, Boroumand-Noughabi S, Ferns GAA (2018). Down-regulation of CatSper 1 and CatSper 2 genes by lead and mercury. Environ Toxicol Pharmacol.

[B037] Nikpoor P, Mowla SJ, Movahedin M, Ziaee SAM, Tiraihi T (2004). CatSper gene expression in postnatal development of mouse testis and in subfertile men with deficient sperm motility. Hum Reprod.

[B038] Ostermeier GC, Goodrich RJ, Moldenhauer JS, Diamond MP, Krawetz SA (2005). A suite of novel human spermatozoal RNAs. Andrology.

[B039] Paradowska-Dogan A, Fernandez A, Bergmann M, Kretzer K, Mallidis C, Vieweg M, Waliszewski P, Zitzmann M, Weidner W, Steger K, Kliesch S (2014). Protamine mRNA ratio in stallion spermatozoa correlates with mare fecundity. Andrology.

[B040] Pardede BP, Agil M, Karja NWK, Sumantri C, Supriatna I, Purwantara B (2022). PRM1 gene expression and its protein abundance in frozen-thawed spermatozoa as potential fertility markers in breeding bulls. Vet Sci.

[B041] Qi H, Moran MM, Navarro B, Chong JA, Krapivinsky G, Krapivinsky L, Kirichok Y, Ramsey IS, Quill TA, Clapham DE (2007). All four CatSper ion channel proteins are required for male fertility and sperm cell hyperactivated motility. Proc Natl Acad Sci USA.

[B042] Ren D, Navarro B, Perez G, Jackson AC, Hsu S, Shi Q, Tilly JL, Clapham DE (2001). A sperm ion channel required for sperm motility and male fertility. Nature.

[B043] Rogenhofer N, Dansranjavin T, Schorsch M, Spiess A, Wang H, Von Schönfeldt V, Cappallo-Obermann H, Baukloh V, Yang H, Paradowska A, Chen B, Thaler C, Weidner W, Schuppe H-C, Steger KS (2013). The sperm protamine mRNA ratio as a clinical parameter to estimate the fertilizing potential of men taking part in an ART program. Hum Reprod.

[B044] Segabinazzi L, Chaves L, Araujo E, Oliveira S, Andrade LP, Okada C, Andrade V, Dell’Aqua C, Papa F, Alvarenga M (2017). Dip quick staining modified for morphological evaluation to equine spermatozoa. J Equine Vet Sci.

[B045] Roser JF, McKinnon AO, Squires EL, Vaala WE, Varner DD (2011). Equine reproduction..

[B046] Sharma R, Agarwal A, Zini A, Agarwal A (2011). Sperm chromatin..

[B047] Steger K, Fink L, Failing K, Bohle RM, Kliesch S, Weidner W, Bergmann M (2003). Decreased protamine-1 transcript levels in testes from infertile men. Mol Hum Reprod.

[B048] Steger K, Wilhelm J, Konrad L, Stalf T, Greb R, Diemer T, Kliesch S, Bergmann M, Weidner W (2008). Both protamine-1 to protamine-2 mRNA ratio and Bcl2 mRNA content in testicular spermatids and ejaculated spermatozoa discriminate between fertile and infertile men. Hum Reprod.

[B049] Tamburrino L, Marchiani S, Minetti F, Forti G, Muratori M, Baldi E (2014). The CatSper calcium channel in human sperm: relation with motility and involvement in progesterone-induced acrosome reaction. Hum Reprod.

[B050] Zhang X, Gabriel M, Zini A (2006). Sperm nuclear histone to protamine ratio in fertile and infertile men: evidence of heterogeneous subpopulations of spermatozoa in the ejaculate. J Androl.

